# Effects of mitoTALENs-Directed Double-Strand Breaks on Plant Mitochondrial Genomes

**DOI:** 10.3390/genes12020153

**Published:** 2021-01-25

**Authors:** Shin-ichi Arimura

**Affiliations:** Graduate School of Agricultural and Life Sciences, The University of Tokyo, Tokyo 113-8657, Japan; arimura@g.ecc.u-tokyo.ac.jp; Tel.: +81-70-3284-6496

**Keywords:** plant mitochondrial genome, mitoTALENs, genome editing

## Abstract

Mitochondrial genomes in flowering plants differ from those in animals and yeasts in several ways, including having large and variable sizes, circular, linear and branched structures, long repeat sequences that participate in homologous recombinations, and variable genes orders, even within a species. Understanding these differences has been hampered by a lack of genetic methods for transforming plant mitochondrial genomes. We recently succeeded in disrupting targeted genes in mitochondrial genomes by mitochondria-targeted transcription activator-like effector nucleases (mitoTALENs) in rice, rapeseed, and *Arabidopsis*. Double-strand breaks created by mitoTALENs were repaired not by non-homologous end-joining (NHEJ) but by homologous recombination (HR) between repeats near and far from the target sites, resulting in new genomic structures with large deletions and different configurations. On the other hand, in mammals, TALENs-induced DSBs cause small insertions or deletions in nuclear genomes and degradation of mitochondrial genomes. These results suggest that the mitochondrial and nuclear genomes of plants and mammals have distinct mechanisms for responding to naturally occurring DSBs. The different responses appear to be well suited to differences in size and copy numbers of each genome.

Mitochondria, in addition to generating cellular energy in the form of ATP through oxidative phosphorylation, also produce reactive oxygen species that cause double-strand breaks (DSBs) in the mitochondrial DNA. DSBs in mitochondrial DNAs can also be created by replication errors [1,2]. DSBs of DNA can be severely damaging, so organisms have developed mechanisms for repairing them. Such mechanisms have also been studied extensively in plant mitochondria [3,4,5]. DSBs are mainly repaired by two mechanisms, homologous recombination (HR) and non-homologous end joining (NHEJ). HR is a high-fidelity repair system that uses an intact copy of the sequence as a repair template, while NHEJ attaches the ends of DSBs, without using templates, and can result in small insertions or deletions (In/Dels) at the repaired sites. 

## 1. Repair Pathways for Naturally Occurring DNA Double-Strand Breaks 

**Figure 1:**
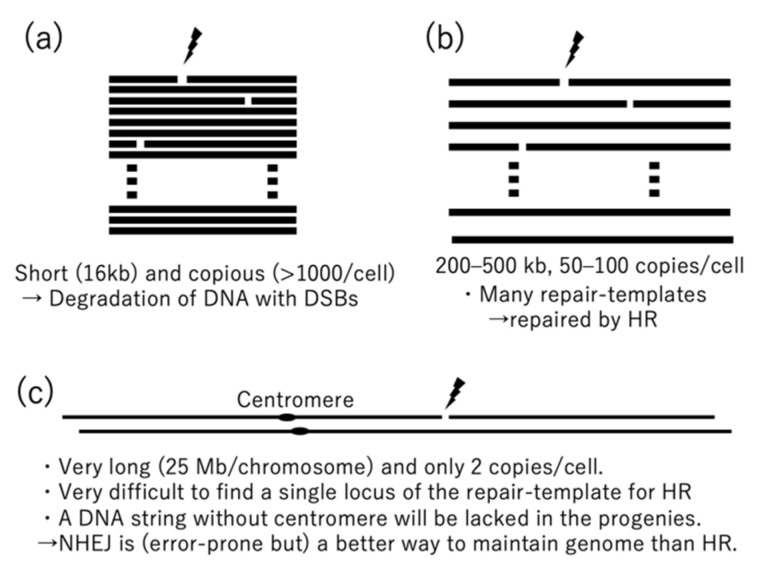
Comparison of double-strand breaks in mitochondrial and nuclear genomes. (**a**) Mammalian mitochondria, (**b**) plant mitochondria, and (**c**) mammalian and plant nuclei.

In the nuclei of plants and mammals, NHEJ is a major pathway for DSB repair (although it is not a major pathway in meiosis and HR is major even in somatic cells in the moss *Physcomitrella patens*). NHEJ is well-suited for the nucleus because it does not need repair templates, which are rarely available in the nucleus (Figure 1c). Nuclear genomes have a copy of the correct template in the sister chromatid, but the sister chromatid is so large (about 30 Mb in *Arabidopsis*), the template is difficult to find. Instead, prompt end-joining after DSBs seems to be essential in the nucleus, even though it occasionally causes small errors at the repair sites. That is because one of the DNA fragments created by a DSB will not have a centromere, which is needed to pull the fragment (and its many genes) into the daughter cells during mitosis.

DNA repair mechanisms of mitochondria are different from those in the nucleus, and they are also different between plants and mammals. The mitochondrial DNAs (mtDNA) of mammals are circular molecules with sizes of about 16 kb. They can be isolated from some mammalian cultured cells with miniprep kits [6]. The mutation rate of mammalian mtDNA is more than 10 times faster than it is in the nucleus [7,8], but the gene order (synteny) is well conserved in diverse species. For example, the gene order is the same in humans and zebrafish, which diverged about 0.4 billion years ago. In contrast, the mitochondrial genomes of flowering plants (angiosperms) have variable gene orders, variable sizes, and a slower mutation rate (about 10 times slower than that in the nucleus) [9,10]. These differences suggest that mitochondrial DNA repair mechanisms are also different in mammals and plants. In mammalian mtDNAs, linear DNAs generated by DSBs seem to be promptly degraded rather than repaired [11,12,13]. Degrading seems safer than repairing because it is less likely to introduce mutations and because mammalian cells have high (>1000) copy numbers of mtDNAs [14], which makes repair unnecessary.

The mitochondrial genomes of angiosperms are generally much larger than those in mammals. They usually range from 200 kb to 500 kb [10], but can be as low as 70 kb [15] and as high as 12 Mb [16]. Plant mitochondrial genomes are mainly detected as linear molecules, but they can also exist in branched forms and in the form of a small number of circular molecules [17,18,19,20]. However, assembled mtDNAs are usually represented as a small number of circular molecules. *Arabidopsis* cells each have about 300–500 mitochondria, but only about 50–100 mtDNA copies [21], resulting in a shortage of genomic information in most mitochondria. The shortage is compensated for by constant fission and fusion of mitochondria [22,23,24,25]. In the mitochondrial genomes of flowering plants, homologous recombinations (HRs) occur between large identical repeats, resulting in alternative configurations of the genome that are referred to as multi-partite structures [26,27,28]. Large repeats (>500 bp) are more identical and more active in HR than intermediate repeats (500 bp >50 bp) and small repeats (<50 bp) [5,10]. If a DSB naturally occurs in a plant mtDNA, HR seems to be a reliable and reasonable repair method because the DSB ends can easily find template sequences in the mitochondrial genome due to its many copies in each cell and tiny size compared to the nuclear genome (1/300 the size in *Arabidopsis*). They imply that in plants, mtDNA is sufficiently abundant for a repair template to be easily found, but not enough for degradation to be a safe solution.

## 2. mitoTALENs-Directed DSBs in Mammalian and Plant Mitochondrial DNAs

**Figure 2:**
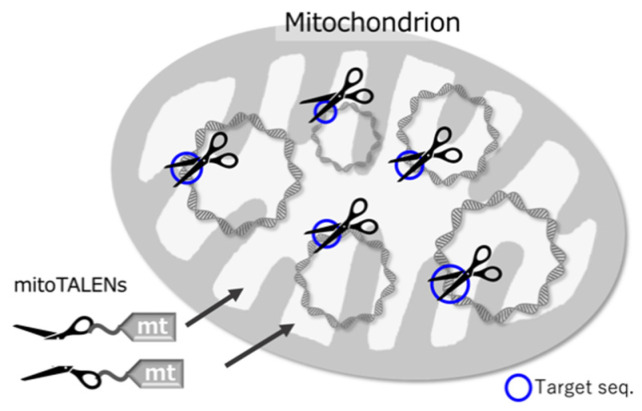
Schematic drawing of mitoTALENs in a mitochondrion. The mitoTALENs are introduced by a mitochondrial targeting sequence (mt), which is cleaved on entry. Each mitoTALEN can be represented as one scissor blade. When joined at the target site, they form a functional scissor-like nuclease.

Different sequence-specific nucleases, including CRISPR/Cas9, transcription activator-like effector nucleases (TALENs), and zinc finger nucleases (ZFNs), are frequently used to disrupt targeted sites in nuclear genomes [29,30,31]. The CRISPR/Cas9 system is used in most nuclear genome editing [31,32], but it doesn’t work well with mitochondrial genomes because it is difficult to transport the guide RNAs that CRISPR/Cas9 uses into the mitochondria [33]. On the other hand, TALENs recognize DNA sequences through an assembly of protein components, and thus TALENs can be easily and effectively transported into mitochondria by merely attaching a mitochondrial pre-sequence to their N-terminus. Therefore, TALENs are now mainly used for creating DSBs at specific sites in mitochondria (Figure 2). TALENs are usually used as a pair, with each molecule recognizing 10–20 bp sequences before and after a 10–20 bp target sequence. Each molecule, in addition to its recognition domain, has a FokI nuclease. After a sequence is recognized, the FokI domains of the paired molecules dimerize and cut out the targeted sequence.

**Figure 3:**
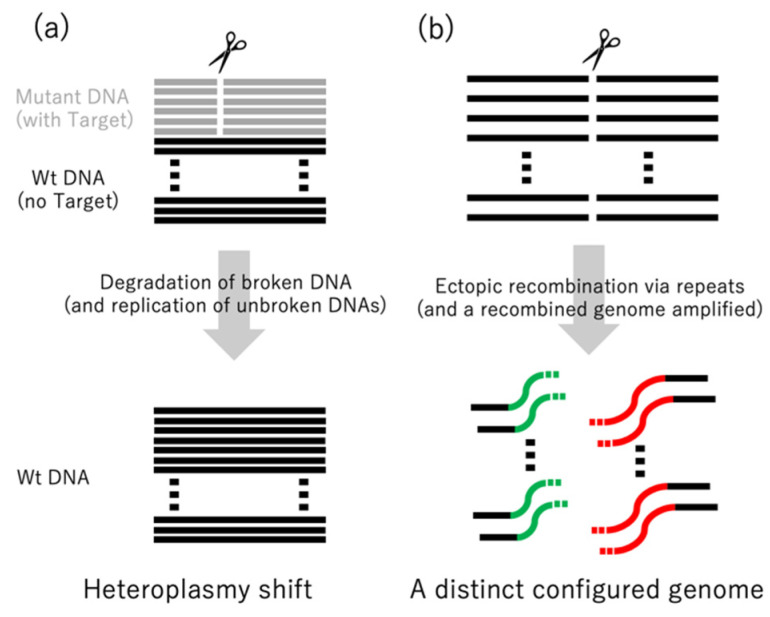
Comparison of the effects of mitoTALENs in mammalian and plant mitochondria. In mammals (**a**), mitoTALENs create DSBs at different sites of mutation, resulting in selective elimination of the mutant DNAs (resulting in a shift from heteroplasmy to homoplasmy). In plants (**b**), all copies of the specific sites are cut, resulting in different conformations of the genome by recombinations between repeats near and far from the target sites.

mitoTALENs, i.e., TALENs with mitochondrial targeting peptides (Figure 2), were first used in mammalian cultured cells [34] and then in mice [35]. In these and other cases, the targeted sequences were located in heteroplasmic mitochondrial DNAs. Heteroplasmy refers to the presence of two or more kinds of mitochondrial DNAs in a cell, which may have different SNPs or In/Dels. Heteroplasmy is observed in healthy mammalian cells and can also increase in an age-dependent manner [8]. Some mitochondrial diseases are characterized by heteroplasmy that occurs as a result of harmful mutations. When the ratio of mutated DNAs rises above a threshold (60–90%), defects in respiration and other symptoms can occur [8,36,37]. Decreasing the ratio of mutated mtDNAs is thought to heal the mitochondrial diseases. In these cases, mitoTALENs can be useful as they could target and destroy the mutated DNAs. This has been demonstrated in cultured cells from humans and mice with pathogenic mtDNA mutations. mtDNAs with the target sequences were specifically decreased (Figure 3a), and respiration-defective cells were rescued (reviewed in [8]). In mammalian cells, mtDNAs with DSBs are quickly degraded rather than repaired [13]. The degradation is carried out mainly by the exonuclease activity of DNA polymerase γ [11,12]. 

We recently made plant expression vectors for pairs of mitoTALENs on Ti-plasmids and introduced them into the nuclei of BT-type cytoplasmic male sterility (CMS) rice and Kosena-type CMS rapeseed [38]. We targeted two genes (*orf-79* and *orf-125*) that were thought to be responsible for CMS in these plants. CMS is an agriculturally important trait; CMS varieties are useful because they can produce F1 hybrid seeds easily and efficiently. In both rice and rapeseed, many of the transformants had large (several hundred bp to several kb) deletions in their mitochondrial genomes that included the target genes. The ends of the remaining sequences were connected not to each other’s ends but to sequences at distant loci (Figure 3b). The connections occurred through repeats that ranged in size from 10 to 1000 bases and that had identities as low as 90%. The recombinations altered the configuration of the genome by introducing or deleting repeats and changing the gene order. We also used mitoTALENs to knock out two redundant mitochondrial genes (*atp6-1* and *atp6-2*) in *Arabidopsis*, which resulted in similar alterations of the genome [39]. Homologous recombinations usually faithfully repair DSBs by using templates. However, repeated attacks of mitoTALENs on the target site might create a shortage of templates, resulting in recombinations via intermediate and short repeats. Such ectopic recombinations are usually suppressed by factors like MSH1 and other repair mechanisms [4,5]. Several lines of rice, rapeseed, and *Arabidopsis* were generated by transformation with mitoTALENs. The same repaired genome structures were detected in each of the lines, indicating that the mitochondrial genomes of each plant were in homoplasmic states.

The above studies demonstrate how sequence-specific nucleases bring about different outcomes in the mitochondrial and nuclear genomes of plants and mammals. The difference in the outcomes can be attributed to distinct repair mechanisms that are specifically adapted to each genome.

## Data Availability

Not applicable.
